# Primary and Community-Based Home Care Services for Older Adults: A Scoping Review of Eligibility Criteria and Homebound Status

**DOI:** 10.3390/healthcare14213670

**Published:** 2026-10-28

**Authors:** Dimitra Iosifina Papageorgiou, Stella Ploukou, Nikolaos Vlachopoulos, Anna Bettina Haidich, Emmanouil Smyrnakis

**Affiliations:** 1 Laboratory of Primary Health Care, General Practice and Health Services Research—Medical School, Aristotle University of Thessaloniki, 54124 Thessaloniki, Greece; ddpapage@auth.gr (D.I.P.); sploukou@auth.gr (S.P.); 2 251 Hellenic Air Force General Hospital, 11525 Athens, Greece; vlacniko@gmail.com; 3 Laboratory of Hygiene, Social and Preventive Medicine, and Medical Statistics, Aristotle University of Thessaloniki, 54124 Thessaloniki, Greece; haidich@auth.gr

**Keywords:** ageing, home care services, home health care, homebound persons, primary health care, community health services, community-based care

## Abstract

**Highlights:**

**What are the main findings?**

- Eligibility criteria for home-based care, based on cognitive status, functional dependency or frailty, vary substantially and are inconsistently defined across studies.
- Although homebound status was reported in only a few studies, applying a purpose-built classification framework suggested that many more studies may involve homebound or at-risk populations, a pattern extending beyond frailty-related groups.

**What are the implications of the main findings?**

- Eligibility criteria, as currently reported, do not consistently identify homebound status.
- Future research should examine whether a validated primary care screening tool can identify homebound risk earlier.

**Abstract:**

Background/Objectives: Home-based primary care and community health services can facilitate ageing in place. However, the eligibility criteria for these services vary widely, and how consistently homebound status is identified remains unclear. This scoping review explored the existing literature on primary care or community care home-delivered services for adults aged 60 and older. This paper, the first of two reporting findings from the same review protocol, examines the eligibility criteria used to identify candidates for such care and the extent to which study populations meet homebound criteria. Methods: A scoping review of MEDLINE, Cochrane Central Register of Controlled Trials, Scopus, ClinicalTrials.gov, ProQuest (grey literature) and hand-searching of the citation list was conducted up to February 2026. The study protocol was prospectively registered. Reporting followed the PRISMA-ScR guidelines. Results: We screened 2834 records (initial and updated searches) and 64 studies published between 1990 and 2026 were included. Eligibility criteria varied substantially, most commonly based on cognitive status, functional dependency, or frailty, though inconsistently defined. Homebound status was rarely reported (11 studies). Applying a classification framework developed for this review, an additional six studies were classified as possibly homebound and 27 studies as at-risk of homebound, without being labelled as such, a pattern extending beyond frailty-related groups to psychosocially vulnerable and general older populations. Conclusions: Eligibility criteria for home-based care are inconsistently defined and reported. Homebound status is rarely reported, even in populations that appear to meet homebound or at-risk criteria. Future research should examine whether a validated primary care screening tool can identify homebound risk earlier.

## 1. Introduction

The ageing population is rapidly increasing worldwide, and by 2050 the number of individuals aged 60 years and older is expected to double and reach 2.1 billion (from 1 billion in 2020), while the population aged 80 or older is expected to triple from 2020 to 2050 and reach 426 million in 2050 [1,2]. The demographic changes expected in the following decades, with the transition towards longer life expectancy in combination with the progression of medicine in the management and control of chronic diseases, lead to the need to adjust healthcare services to meet the needs of an ageing population. This demographic shift presents substantial challenges for health systems, especially considering that within the ageing population there is also a portion of older adults who cannot easily access healthcare services due to physical, cognitive, social, or environmental limitations. This represents a community-dwelling “invisible” population, known as homebound, whose complex needs necessitate home visits to be understood, foreseen, and addressed by providers. Considering the multiple definitions and different causes of confinement, it has been challenging to estimate the percentage of this population, which in studies varies between 5% and 65% [3,4,5,6].

The homebound population is highly heterogeneous, and there are multiple definitions in the literature [7]. Definitions range from administrative criteria, such as the United States Medicare criteria [8] to a concept analysis describing six attributes, among which life-space confinement and the need for help to leave the home [9] and other approaches use the frequency of leaving home [4,10]. Most definitions use a combination of these factors [7]. More details on definitions are presented in Appendix C.

Eligibility criteria for home-delivered care go beyond the homebound. Different programmes and health systems use different thresholds for age, functional dependency and multimorbidity to decide who qualifies, with some targeting people at high risk of institutionalisation and others drawing from the general older-adult population [11,12]. This variation has been reported even between neighbouring regions within the same country, not just across countries [13], making the comparison of study populations across literature difficult. Beyond where thresholds are set, it is unclear how consistently these criteria are defined, measured and reported in the literature describing target populations for home-based care, a gap examined in this review.

The delivery of primary-care home-based interventions and community health services at home has emerged as a valuable response to the complex and multiple needs of the older population, offering preventive, curative, rehabilitative, and palliative care while also integrating social care [14,15]. When adapted to healthcare system characteristics, they can support ageing in place, quality of life, and independence, and reduce the need for institutional care [16,17]. However, the organisation of primary care and community health services in the home has important variation across different settings and populations, making comparisons complicated [16,18]. Fragmented, poorly coordinated care may result in services that do not meet users’ needs [19,20], although meeting these needs is a key indicator of service quality [21,22,23]. Unmet needs are common among home care recipients and include various domains such as health-related, social and practical aspects [18,20,24,25], including assistive technologies in order to support individuals with disabilities in performing daily living activities [26]. Frail older people living alone and more marginalised populations tend to have more complex needs [27] and frequently these are related to personal care, care-giver support, adult day care or transportation [23]. The way these needs are assessed and how well home-based services address them is examined in a second part of this review. This paper focuses on a first step toward meeting those needs. It examines how eligibility criteria define the population these services are intended for, and whether homebound status is defined.

Considering the variability of recipients’ needs and the variety of characteristics used to identify a home-confined patient, it is difficult to compare findings across studies or establish which home-delivered care models are suitable matches, for whom, and under what conditions. Part of this inconsistency may stem from the heterogeneity of the homebound population itself, whose reasons for confinement are diverse, and part from the presence of de facto homebound individuals within study populations who are never formally identified as such. Previous reviews have focused on how homebound status is defined in studies of people already identified as homebound [6,7]. Less attention has been given to the wider literature on home-based care, where study populations may share homebound characteristics without being described as such.

To help move toward more standardised, person-centred models of home-based care, this study reviewed the literature on primary care and community health services delivered at home to adults aged 60 years and older. This paper focuses on two interconnected areas: (1) the eligibility criteria used to select candidates for home-delivered care and (2) whether homebound status was identified in the study population and whether the population in practice met homebound criteria, even when not formally labelled as such. A second part of this review reports on how the health needs of this population were assessed and the extent to which those needs shaped the design and delivery of services.

## 2. Materials and Methods

### 2.1. Design

The scoping review study protocol was developed using the five-stage methodological framework proposed by Arksey and O’Malley [28] and further refined by the Joanna Briggs Institute [29]. Findings were reported according to the PRISMA Extension for Scoping Reviews (PRISMA-ScR) reporting guidelines (Supplementary File: PRISMA-ScR checklist) [30]. The final version of the protocol was revised by all research members and was prospectively registered with the Open Science Framework (OSF) on the 2nd of March 2025 (DOI: 10.17605/OSF.IO/8JFYM).

### 2.2. Identifying Research Questions

The PCC framework (population, concept and context) guided the identification of the research questions [29]. This paper, the first of two reporting findings from a single review protocol, aimed at answering the following questions:

1. What eligibility criteria are used to identify adults aged 60 years and older as candidates for receiving primary care services at home?
2. When the term “homebound” or the term “confined to the home” is used as a characteristic describing the study population to be included in the study, how is it used and what are the distinctive characteristics of the term?

To answer the second question more fully, the characteristics identified in studies that used the term “homebound” or the term “confined to the home” were also used to examine study populations that were not described as homebound, but showed similar characteristics (Section 3.3 and Appendix C).

### 2.3. Identifying Relevant Studies

To identify potentially relevant studies, the following databases were used: MEDLINE (PubMed), Cochrane Central Register of Controlled Trials, Scopus, ClinicalTrials.gov and ProQuest (grey literature). The search strategy was developed for MEDLINE, reviewed by all the team members through discussion and adapted to the other databases (Appendix A). The search was carried out from the inception of each database until the date the initial search was run (11 March 2025). An updated search was conducted from 11 March 2025 to 23 February, 2026, to identify any additional studies published within the previous year. Reference lists of the studies included were hand-searched for additional sources, as specified in the protocol. In addition, the hand search was extended to all studies assessed at the full-text screening stage. All identified citations were exported to Mendeley Reference Manager (Mendeley Ltd., London, UK).

For this review, participants living at home and aged 60 years and older were included to broaden the study population and capture more individuals who potentially qualify to receive home-based care. Studies without a reported minimum age of 60 were included only when the service was designed for older adults or an ageing population. Studies focusing on the development or delivery of home interventions aimed at improving population health were included. We included studies in which the intervention or service was delivered by primary healthcare professionals, community care staff, or case managers. Although the exclusion criteria were not designed to restrict studies based on specific health needs, interventions related to emergency care for potentially immediate life-threatening conditions and post-operative home visits aimed at supporting patients immediately after hospital discharge and conducted within three months of surgery (late post-operative period) were excluded [31]. No language restrictions and no limitations on the geographic region of the study population were applied to broaden the search. These eligibility criteria were set for the full review protocol, and both papers draw on the same set of included studies. This paper examines how these studies defined their target populations and used the homebound term, while the second paper addresses need assessment and how home-based services are developed according to assessed needs. More details about the eligibility criteria are presented in Table 1.

### 2.4. Study Selection

After removal of duplicates, all citations were entered into the Rayyan application (Rayyan Systems Inc., Cambridge, MA, USA). Citations were screened manually, following a two-stage study selection process performed independently by two reviewers (DIP and NV). A title/abstract screening tool using eligibility criteria was developed to facilitate and standardise the title/abstract screening (Appendix B) [32]. At the full-text screening stage, the same two reviewers (DIP and NV) independently conducted a full-text review to determine whether each included study met the inclusion criteria. Full texts published in languages other than English were assessed after machine translation into English (Google Translate, Google LLC, Mountain View, CA, USA), including Supplementary Material where available. Since the primary aim of the review was to map participant characteristics and health-needs assessment procedures or tools, studies were eligible for inclusion irrespective of whether data analysis was completed. Reasons for exclusion of full-text studies were recorded. During both stages, pilot testing on randomly selected articles was conducted until a minimum of 80% agreement was achieved between the reviewers. Disagreements were resolved at both stages with discussion between the two reviewers and, if necessary, by a third reviewer to reach consensus. Inter-rater agreement was assessed during both stages to ensure consistency.

### 2.5. Charting the Data from the Included Studies

#### 2.5.1. Data Items

We extracted the following data:

1. Study characteristics (country, year of publication, study design, main focus of the intervention, involved healthcare professionals);

2. Target population (population characteristics and eligibility criteria);

3. Homebound status (use of the term “homebound”/“confined to the home” as an eligibility criterion and the use of other indicators related to the homebound status characterising participants);

4. Health-needs assessment (assessment approach, assessed needs domains, tools/instruments, the relationship between the needs assessment, and the intervention design, including if it informed the delivery of the intervention and which needs were matched to a service);

5. Gaps in the studies related to the aims of the review.

This paper reports findings on target population characteristics, eligibility criteria and homebound status. Findings on health-needs assessment and the alignment between assessed needs and service delivery are reported in a second part of this review.

#### 2.5.2. Data Extraction Process

A standardised data extraction form guided by the research questions and guidance for the use of the form were developed by the reviewer team (DIP, NV, SP) to extract the relevant data from the included studies (Appendix C). The data extraction form was piloted on a random sample of ten eligible studies to assess the consistency of the extraction process and the suitability of the extraction form. The form was modified based on the reviewers’ comments, with particular attention given to redundant or missing data and to points requiring clarification. Data were extracted by the primary reviewer (DIP) and verified by a second reviewer (SP), as described below. Discrepancies were discussed with a third reviewer (NV). Where Supplementary Information on the characteristics of the intervention was required, the study protocol was reviewed to retrieve the relevant details. Risk of bias of the included studies was not assessed [29].

### 2.6. Collating, Summarising and Reporting the Results

An initial codebook, guided by the research questions, was developed by the primary reviewer (DIP) and iteratively revised through engagement with the included studies (Appendix C). During this process, it was observed that many studies described populations with homebound-consistent characteristics without using the term per se. The extraction and coding approach was adjusted accordingly, as anticipated in the protocol, leading to the development of a classification framework to assess the extent to which study populations met homebound characteristics. An indicator was coded only when it applied to all or the great majority of participants, as reported in the eligibility criteria, target population description or baseline characteristics. Codes were added, merged or redefined as needed, with studies coded under earlier versions of a definition re-checked once it was finalised. Verification of the finalised codebook was applied at two levels, depending on whether a coding scheme required interpretive judgement about study content. For descriptive information (e.g., sample size, involved healthcare professionals, eligibility criteria), data extracted by the primary reviewer were independently verified against the source publications by the second reviewer (SP) across all included studies. Discrepancies were identified and resolved through discussion. For codes also requiring interpretive judgement (e.g., indicators characterising participants’ homebound status), the finalised codebook was independently applied by the second reviewer to a random subsample of 25% of the included studies (*n* = 16), stratified by main intervention rationale [33]. Agreement was quantified using percentage agreement and Cohen’s kappa [34], with disagreements resolved through discussion. Results were synthesised narratively, using frequencies and content analysis [35].

## 3. Results

**Figure 1:**
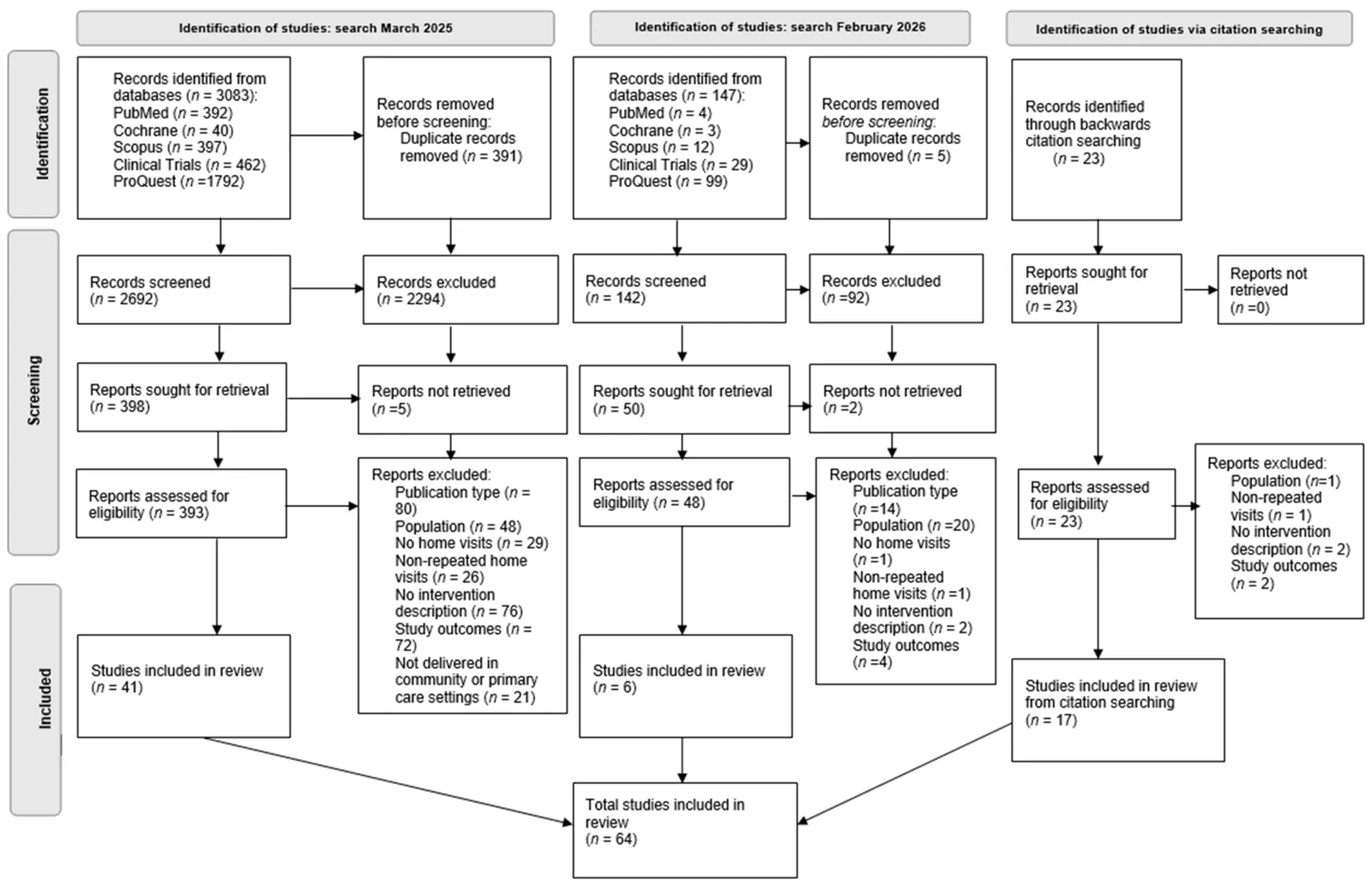
PRISMA flow diagram.

The literature search yielded a total of 3083 records, of which 391 were removed as duplicates (Figure 1). After title and abstract screening, 2294 records were excluded, leaving 398 studies for full-text retrieval. Five records could not be retrieved and at full-text screening, 352 records were excluded with reasons. A total of 41 articles were eligible and subsequently included. Additional records were retrieved through citation searching and 17 articles were included. The updated search identified 147 additional records, of which five were duplicates. Of the 142 records screened, 92 were excluded and 50 reports were sought for full-text retrieval. Two could not be retrieved, and of the 48 reports assessed in full text, 42 were excluded, leading to the inclusion of six additional studies. A total of 64 records were included in the review.

### 3.1. Country of Origin and General Characteristics

Most of the studies were conducted in the United States (*n* = 16, 25.0%), followed by the Netherlands (*n* = 9, 14.1%) and Canada (*n* = 9, 14.1%). Four studies were conducted in rural or remote areas [36,37,38,39]. Included studies were published from 1990 to 2026 with the majority (*n* = 51, 79.7%) being published from 2010 onwards, reflecting the growing research attention in this model of service for the ageing population. Half of the studies were randomised controlled trials (*n* = 32, 50.0%) (Table 2). The total number of participants was 17,561. The sample size of the included studies ranged from 3 to 1147 participants, with a median sample size of 168 participants and in 76.6% of studies (*n* = 49) participants were predominantly female. Mean age of patient populations ranged from 67 to 88.1 years across 45 studies, while median age (reported in three studies) ranged from 72.5 to 83.5 years. In six studies age was reported in predefined categories (e.g., 60–69 years), five studies reported age only through an eligibility threshold (e.g., ≥65 years), and five studies provided no age information except from a broad description of the population as older or elderly adults (Table 2). This heterogeneity in how age was reported precluded the combination of data into a single, consistent age measure across studies.

Six categories of intervention focus emerged inductively from the included studies (Table 2). In 24 studies (37.5%) the provision of care coordination or the integration of services was the focus and intervention mechanism. Preventive and early-detection interventions, typically structured around comprehensive geriatric assessment and proactive home visits, were the second most common study rationale (*n* = 15, 23.4%). Ten studies focused on the disease/symptom management (15.6%), followed by studies focusing on functional independence and reablement (*n* = 7, 10.9%), on the provision of psychosocial support (*n* = 4, 6.3%) and palliative/end-of-life care (*n* = 4, 6.3%). Within the disease/symptom management category, dementia and cognitive impairment represented the largest subgroup (*n* = 4) [41,42,43,79], followed by medication-related interventions (polypharmacy management and medication review) (*n* = 4) [37,38,50,92]. The remaining studies addressed multimorbidity (*n* = 1) [67] and cardiorespiratory chronic disease (*n* = 1) [66].

Twenty-seven studies combined more than one rationale in their design [36,39,40,41,42,47,48,50,53,55,56,57,58,60,66,68,69,73,74,76,77,79,88,92,95,96,98]. Early identification of risks and a proactive identification of unmet needs with coordinated service provision as the response to those needs was the most common combination found in ten studies [39,40,55,56,58,60,69,88,95,98]. Of these ten, five were primary care-based: the CCFE (Care Chain Frail Elderly) aimed at patients with complex needs registered at a General Practitioner practice [55], the primary-care ACP (Anticipatory Care Planning) intervention [95], the U-PROFIT (Utrecht PROactive Frailty Intervention Trial) [56], the [G]OLD home visitation programme for the early detection of health and wellbeing problems in ‘‘apparently healthy” seniors [60] and the Geriatric Care Model [58]. The remaining five were community-based, linked to primary care services as part of the coordination model: the CARE model (Coordination and Advocacy for Rural Elders) for frail older veterans living in remote underserved areas [39], the HCOACDM (High-Need Community-Dwelling Older Adults Care Delivery Model) [98], the AIMS (Age-Friendly Integrative Mobile Services) [40], the Eldercare project for the identification of unmet needs of older adults living independently in the community [69] and the C-HSPP (community-based health–social partnership programme) aimed at improving self-care [88].

Across the 64 studies, registered nurses or nurse practitioners were the most consistently involved profession (*n* = 60, 93.8%) [36,39,40,41,42,43,44,45,46,47,49,50,51,52,53,54,55,56,57,58,59,60,61,62,63,64,65,66,67,68,69,70,71,72,73,74,75,76,77,78,79,80,81,82,83,84,85,86,87,88,89,90,91,92,93,94,95,96,97,99], followed by physicians (general practitioners or geriatricians (*n* = 48, 75.0%) [36,37,38,39,41,44,45,46,49,51,52,53,54,55,56,58,59,60,61,62,63,64,65,66,67,68,69,70,71,72,73,75,76,79,80,81,82,83,84,87,90,91,92,93,94,95,96,97], social workers and providers (*n* = 32, 50.0%) [36,39,41,42,43,44,45,46,48,49,51,52,54,55,64,66,67,71,74,81,83,84,86,88,89,90,93,94,95,96,97,98], occupational therapists (*n* = 22, 34.4%) [36,43,44,45,47,55,59,60,64,65,68,72,74,76,77,78,81,83,84,87,91,93] and physiotherapists (*n* = 21, 32.8%) [36,44,45,55,59,60,65,68,72,73,74,76,78,81,83,84,85,87,91,93,95]. In 20 studies the role of case manager or care coordinator was reported (31.3%) [36,42,45,46,51,59,66,67,68,73,74,75,84,86,88,89,94,96,97,98]. Other specialties such as pharmacists (*n* = 17, 26.6%) [36,37,38,40,45,49,50,51,55,58,59,66,67,79,92,93,95], mental health professionals (*n* = 11, 17.2%) [36,52,67,76,77,79,81,90,93,94,97], dietitians (*n* = 6, 9.4%) [45,64,67,76,81,83] and community health workers/ community care providers (*n* = 4, 6.3%) [45,88,89,93] were less often involved.

When classified by overall team structure, 34 studies (53.1%) [40,42,46,49,50,51,53,55,56,57,58,60,61,62,63,66,67,69,70,73,74,75,79,80,82,83,84,87,88,89,92,94,95,99] were nurse-led, most commonly by a community, district or advanced practice nurse coordinating the assessment, care planning and follow-up. In 20 studies (31.3%) [36,37,39,41,43,47,52,59,64,68,76,77,78,81,86,90,91,93,96,97] the intervention team was multidisciplinary and there was no evident specialty exclusively leading the intervention. Physician-led home-based interventions were comparatively rare (*n* = 5, 7.8%) [44,54,65,71,72], as were interventions led by other single disciplines like social workers (*n* = 2) [48,98], community health workers (*n* = 1) [45], rehabilitation therapists (*n* = 1) [85] and pharmacists (*n* = 1) [38].

The case manager role, which was recognised in 20 studies, was mostly present in studies focusing on care coordination/integration (*n* = 13) [45,51,68,73,74,75,84,88,89,94,96,97,98] consistent with case management being a structural mechanism for coordinating care across providers and settings. However, case manager roles also appeared in studies focused on disease/symptom management (*n* = 3) [42,66,67], functional independence/reablement (*n* = 1) [59], palliative/end-of-life care (*n* = 2) [36,46] and studies focusing on prevention/early detection (*n* = 1) [86]. Where reported, the professional background of the case manager was usually nursing (*n* = 13) [42,45,46,51,59,73,74,75,88,89,94,96,97], followed by dual-discipline backgrounds in nursing and social care [84], backgrounds in social work [98] and non-clinical professions [67].

### 3.2. Target Population and Eligibility Criteria

The studies targeted eight population characteristics (Table 3). The most frequent population characteristic was frailty (*n* = 20, 31.3%). The second and third largest categories were functional decline (*n* = 11, 17.2%) and multimorbidity/complex chronic conditions (*n* = 10, 15.6%), respectively. Specifically, in the functional decline group, two studies [63,93] focused on participants at high risk of falling and mobility impairment, while three studies [44,54,71] targeted homebound participants due to functional dependency. In the multimorbidity group, four studies [36,46,49,66] specifically addressed palliative or advanced care planning needs due to life-limiting conditions. Eight studies (12.5%) included the general population using only the criteria of the age threshold and living in the community. Other studies targeted participants with psychosocial vulnerabilities (*n* = 4, 6.3%) (e.g., marginally housed people [52], low-income people [48], populations living in underserved areas [40] or living alone [99]), participants with medication-related risks (*n* = 3, 4.7%) and frequent service users (*n* = 2, 3.1%). Six studies on patients with dementia or cognitive impairment [41,42,43,76,77,79] and a study on frail patients [87] were characterised by the dual recruitment of the family caregiver.

A minimum age was specified as an eligibility criterion in 47 studies (73.4%). It was 60 years in 11 studies [48,50,52,56,61,76,77,78,81,88,89], 65 years in 17 [40,44,47,51,67,68,73,74,75,80,86,91,92,94,96,98,99], 70 years in seven [57,59,63,64,70,79,95], 75 years in eight [39,53,60,62,72,83,90,93] and 80 years in four [38,65,69,82]. In the 11 studies with a minimum age of 60, mean ages ranged from 70.7 to 81.9 years where reported. In three of these studies, the youngest participants were aged 60 to 64 [76,77,88], but no study reported how many participants fell within this age group. The remaining studies described services designed for older people, but identified participants through a clinical condition or service use, either without an age criterion (*n* = 13) [36,37,41,43,45,49,54,55,58,66,71,84,87] or with a broader adult age limit (*n* = 4) [42,46,85,97]. Where reported, mean or median ages in these studies ranged from 67 to 87.5 years. Higher age limits were more common in preventive and early-detection studies, where 11 [53,60,62,63,69,70,82,83,90,93,95] of 15 required participants to be 70 years or older. No clear pattern was observed by country [76,77,88].

Beyond age, the eligibility criteria mostly used across the included studies were cognitive status (*n* = 32, 50.0%), functional status (*n* = 27, 42.2%), frailty (*n* = 23, 35.9%), multimorbidity (*n* = 12, 18.8%), and polypharmacy (*n* = 7, 10.9%) (Table 4). Cognitive status, functional status and frailty were typically applied by requiring either the presence or the absence of the characteristic, effectively using it to characterise the target population’s functional status in either direction. Cognitive status was used as an inclusion criterion in studies targeting patients living with dementia (*n* = 6) and as an exclusion criterion in studies requiring cognitively competent participants (*n* = 11) and was also used as a marker of participants’ consent capacity and literacy or as part of a broader frailty assessment (*n* = 15). Considerable heterogeneity was noticed in how the cognitive status criterion was applied, driven by a wide range of different measures and thresholds used to define it across studies. Within the inclusion group, dementia status was established through a clinical diagnosis alone or combined with a staging classification or a behavioural instrument such as the Neuropsychiatric Inventory Questionnaire (NPI-Q). The exclusion group relied mostly on standardised screening tools (MMSE: Mini Mental State Examination, MoCA: Montreal Cognitive Assessment, Short Portable Mental Status Questionnaire: SPMSQ), although four studies excluded participants based on clinical judgement without naming an instrument [47,53,69,78]. The most heterogeneity appeared in the group where cognitive assessment was used to assess consent or decision-making capacity, sometimes with a proxy substituting when needed, to set a literacy threshold for completing study procedures or, less often, as one item within a broader frailty or risk-assessment tool.

Most studies using a functional status eligibility criterion required the presence of functional dependency (*n* = 25), while two studies required functional independence [53,69]. Ten studies reported a concrete, quantified threshold, such as impairment in one or more Activities of Daily Living (ADL) or impairment in two or more Instrumental Activities of Daily Living (IADL), without specifying the tool name and if dependence on particular abilities was needed to reach that threshold (e.g., bathing, dressing, mobility, meal preparation, etc.), creating heterogeneity in what the same numeric criterion actually captured from study to study [41,47,48,51,73,74,75,77,78,83]. Eight studies defined dependency through homebound status [44,46,49,52,54,65,71,85], which are described in more detail below. Only three studies referenced a validated functional instrument (Outcome and Assessment Information Set: OASIS [50], Contact Assessment plus: CA+ [94] or the Sickness Impact Profile) [63], while the remaining four studies used no specific tool or scale, relying instead on a general description of functional decline [37,70,84,91].

Although 20 studies described targeting the frail population (Table 3), only seven of these used a named or validated frailty tool or scale. The remaining 13 instead relied on clinical judgement and a combination of other domain-specific markers, such as functional dependency, multimorbidity, or polypharmacy, without a specific instrument (Table 5). Three additional studies used validated frailty tools even though their target population was not described as frail. PRISMA-7 was used by Brazil et al. (2022) [95], who recruited participants at risk of functional decline, while Wong et al. (2020) [89], used the Clinical Frailty Scale (CFS) to select non-frail participants from a general older population. The Elder Risk Assessment (ERA) score was used in a population with multimorbidity [49]. In total, 23 studies used frailty as a formal eligibility criterion, and more than half of these (*n* = 13, 56.5%) also required a functional-dependency and/or cognitive-status criterion, reflecting the multidimensional nature of frailty [100]. Most of the validated tools used across these studies (e.g., the Groningen Frailty Indicator: GFI, PRISMA-7, CA+) were purpose-built frailty instruments, while others, like the ERA score, the Probability of Repeated Admission Score (PRA-Plus score), the Dartmouth COOP Functional Health Assessment Charts by the World Organization of Family Doctors (COOP-WONCA) and the frailty index derived from the GP’s electronic records, were broader risk-prediction or functional-status tools adapted to identify patients in a frail condition.

### 3.3. The Term “Homebound” and Its Application

Eleven studies (17.2%) clearly indicated that they were focused on the community-dwelling homebound patient (Table 6). Four studies employed a formal criterion to determine homebound status, most commonly the Medicare homebound definition or a definition that includes Medicare components based on the difficulty and frequency of leaving the house. In the remaining seven studies, homebound status was provided descriptively. In four studies this description appeared within the eligibility criteria used to enrol participants using a combination of functional and clinical dimensions and in three studies the homebound status was described only as a baseline characteristic of participants, with no accompanying definition. In these cases, we examined the eligibility criteria to interpret how homebound status was being used in the study. Three other studies (4.7%) targeted a non-homebound population, restricting recruitment to independent, ambulatory, or cognitively well-functioning older adults. We interpreted these descriptions inversely, to infer the likely profile of a homebound person within each study’s context. The excluded participants were those who were bed-bound or not ambulatory, dependent in daily activities, frail or cognitively impaired, a profile consistent with the dimensions of homebound status described in the literature [7,9].

The remaining 50 studies (78.1%) were evaluated to determine whether the participants displayed characteristics suggesting home confinement or a susceptibility to becoming homebound, based on the details provided in the population description, even though they were not labelled as homebound. To classify these studies, a two-part framework was developed. The first part, Possibly Homebound (PH) indicators, captures more direct signs of confinement, such as needing help to leave home, reported difficulty moving around outdoors or already receiving home-based services. The second part, At Risk of Homebound (ARH) indicators, captures factors that raise the risk of becoming homebound without confirming it, such as frailty, multiple health conditions, or social isolation. A study was classified as PH if it met at least two PH indicators. A study with one PH indicator or with no PH indicator but at least one ARH indicator was classified as ARH. The ARH indicators were designed to favour sensitivity, so that populations characterised by conditions that may lead them to home confinement would not be overlooked. The indicators, their definitions and the decision rules are presented in Table 7, with detailed coding guidance in Appendix C. The two-indicator threshold follows the included studies that defined or described homebound status (Table 6), which usually combined at least two signs, such as a functional limitation together with a care need. Inter-rater agreement was assessed on a stratified sample of 16 studies (25.0%). The two reviewers assigned the same final classification to all 16 studies. Agreement on the individual indicators, in the studies classified with the framework, was 98.3% (115 of 117 indicator decisions, κ = 0.949, 95% CI 0.88 to 1.00) [34]. Of the 50 studies assessed with the framework, 27 were classified as ARH, six as PH and two as “non-homebound” (Table 8). Most of these classifications were supported by more than one indicator. Twenty-four of the 27 ARH studies met at least two indicators and seven of them also met one PH indicator, falling short of the two required for the PH category. Only three studies were classified on a single indicator [64,67,93]. In a sensitivity analysis, lowering the threshold to one PH indicator would have classified 13 instead of six studies as PH. Raising the PH threshold to three indicators would have classified none, as no study met more than two PH indicators. The PH category therefore indicates possible rather than confirmed home confinement. Similarly, the ARH category indicates characteristics associated with a risk of becoming homebound, not that participants will become homebound. Indicators were coded only when they applied to all or most participants, based on the eligibility criteria or baseline characteristics. Each classification therefore describes the overall study population, not individual participants. Nine studies included participants who would fall into different categories (PH, ARH or “not-homebound”). In four studies, the inclusion criteria themselves created separate entry routes or strata associated with different degrees of dependency, so the study population was built to contain more than one severity group and could not be categorised uniformly [75,77,83,97]. In five studies, although participants were recruited using the same pathway and criteria, the resulting sample comprised individuals both with and without functional dependency, varying from “non-homebound” to “possibly homebound” [39,66,76,82,98]. Six studies could not be assigned a homebound-risk classification due to insufficient information. Two of them were implementation or process-evaluation studies. Beyond the eligibility criteria, they reported no participant characteristics, including no functional, frailty or cognitive baseline data [51,58]. The other four studies reported baseline participant information, but not enough to assess the indicators [59,60,73,90].

**Figure 2:**
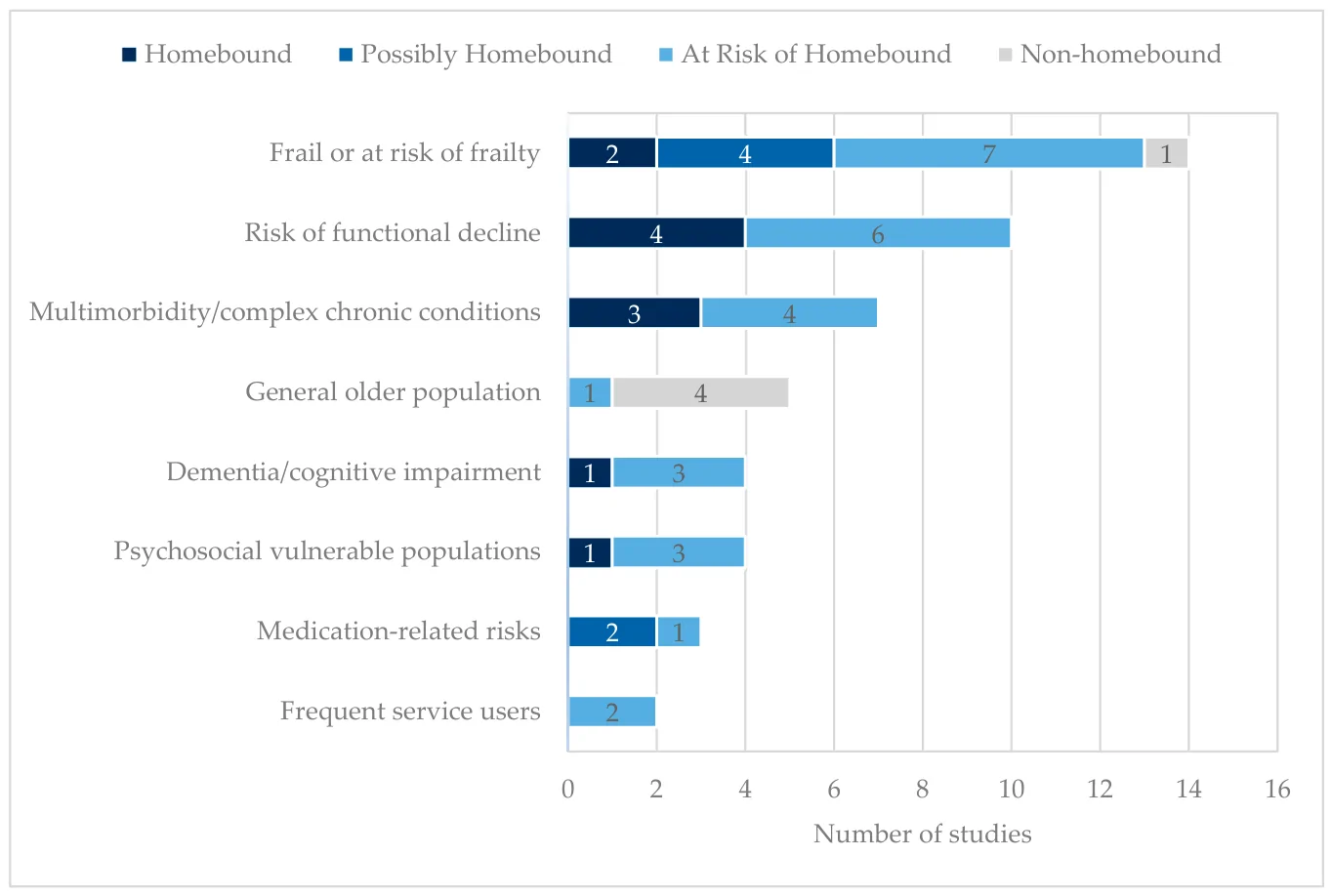
Homebound risk classification by target population group (*n* = 49). Classifications come from two sources: (1) studies reporting their population as homebound (*n* = 11) or non-homebound (*n* = 3) presented in Table 6; and (2) studies classified using the framework developed for this review as possibly homebound (*n* = 6), at risk of homebound (*n* = 27) or non-homebound (*n* = 2) presented in Table 8. Studies with mixed populations (*n* = 9) or insufficient information (*n* = 6) could not be assigned a single category and are not shown.

Overall, of the 64 included studies, 14 reported their population’s homebound status (homebound in 11 and non-homebound in three), 35 were assigned a single category using the framework (six PH, 27 ARH and two non-homebound), and 15 could not be assigned a single category (nine mixed populations and six with insufficient information). Figure 2 illustrates the distribution of homebound-risk classification across the eight target population groups presented in Table 3, based on the 49 studies presented in Table 6 and Table 8. Classification varied by target group. Risk of functional decline and frailty or being at risk of frailty had the highest number of studies classified as homebound or possibly homebound, while the general older population and frequent service users target groups were the only two groups with no homebound or possibly homebound classified studies.

Among the 23 studies that used frailty as an eligibility criterion (Table 5), the homebound classification did not differ clearly by how frailty was assessed. Two of the seven studies that targeted frail patients with a named or validated tool were classified as PH [81,94], as were two of the 13 studies that relied on clinical judgement or unstructured criteria [72,84]. Studies classified as PH or ARH made up a similar share of both groups, four of seven [56,62,81,94] and seven of 13 [50,55,57,70,72,74,84]. Two of the seven studies using a validated tool could not be classified because of insufficient information [58,59].

## 4. Discussion

In this scoping review, 64 primary studies were identified on the delivery of home-based primary care or community health services for adults aged 60 years and older, published between 1990 and 2026. The majority of the studies (*n* = 51, 79.7%) were published from 2010 onwards, a period during which this model of care has expanded gradually, as noted also by Zimbroff et al.’s (2021) systematic review [101]. Most of the interventions aimed at providing coordinated/integrated care to the participants and usually combined it either with a proactive identification of unmet needs and the provision of preventive care or the early identification of potential problems that would impact the ability of participants to live independently at home. Although nurse-led models were most common, multidisciplinary teams were frequently described reflecting the interdisciplinary nature of home-based primary care, as described by Zimbroff et al. (2021) [101]. The role of the case manager was a common structural feature of intervention teams, most common in studies focused on coordination of services and on improving access of care in the community to meet participants’ needs, but it was also present in interventions in which the primary focus was disease management, reablement or palliative care. The involvement of a dedicated case manager/coordinator is recognised in the literature as a response to the complex needs of old people who may benefit from having a consistent point of contact with a professional who could recognise their evolving needs and bridge the gap of care by creating links with the health and social care system [102,103].

Regarding the target population characteristics, studies focused on two broad categories. In some studies, the target group was linked to well-defined clinical diagnoses or markers (e.g., dementia/cognitive impairment, multimorbidity/complex chronic conditions or medication-related risk). In others, the target group was defined by a broader, more graded form of vulnerability. This ranged from more specific situations such as psychosocial conditions (e.g., underserved populations) or functional decline to more multifaceted conditions like frailty, which represents the largest target population group. Finally, a few studies targeted the general population, and in this case, no specific clinical or social criteria were applied, beyond age and community-dwelling status. In these and other preventive studies, a higher age limit was often used to select people at greater risk. In contrast, studies focused on clinical conditions often had no age limit, as the condition itself defined who could be enrolled. Notably, the family caregiver involvement was a consistent feature across all studies targeting dementia or cognitive impairment. This pattern points to the intrinsic dyadic complex nature of dementia in which the person living with dementia and their informal caregiver function as a unit and whose relationship can determine the feasibility and sustainability of any home-based care intervention [104,105].

Eligibility criteria drew mainly on cognitive status, functional dependency and frailty, yet each was defined and measured differently across studies. Cognitive status was the most frequently used criterion, applied to include people living with dementia, to exclude those with cognitive impairment, or to assess capacity to take part, with screening tools and thresholds varying across studies. The implementation of cognitive status as a criterion is linked to a broader concern in geriatrics research [106]. People with cognitive impairment are often excluded, usually through inconsistent screening cutoffs, with little justification and rarely acknowledged as a limitation. This may matter even more in home-based care research, since cognitive impairment is especially common among frail and homebound older adults and excluding these participants risks producing evidence that is less applicable to the population these services are meant to reach. A few studies in our review had a more inclusive approach, allowing a proxy or substitute decision-maker instead of excluding participants outright [84,96].

Studies using functional status, the second most common criterion, usually required the identification of functional dependency but rarely specified which abilities were assessed or which instrument was used. Specifically, 10 studies relied on an ADL/IADL threshold without reporting which tool was used and which specific abilities needed to be impaired to meet it, making comparison across studies difficult. This gap in reporting has been outlined in a systematic review of more than 50 ADL instruments, which found that studies referencing a scale without naming its specific version or items (as they called it a “Katz unspecified” category) scored worse on validity and responsiveness than every properly specified version of the same instrument and that fewer than 10% of reviewed articles described their ADL instrument in enough detail to be evaluated at all [107]. For home-based care specifically, functional decline usually is the main reason someone needs the service in the first place. This lack of specification makes it unclear what threshold of need actually triggers access to care.

Frailty was the largest target group, yet it was identified in very different ways, ranging from validated instruments to clinical judgement alone. Variation also existed in the validated tools, since some of them were specifically frailty instruments (e.g., GFI, PRISMA-7), while others were general risk-prediction or functional-status tools adjusted to frailty (e.g., Elder Risk Assessment, PRA-Plus score, COOP-WONCA) or applied a frailty index directly to already available electronic records. More than half of the 23 studies (56.5%) using frailty as an eligibility criterion required a combination of functional and/or cognitive criteria. This finding is consistent with most frailty tools already including these domains in their scoring. Sun et al. (2024) [108] found that multidomain instruments (e.g., Groningen Frailty Indicator) were more often applied in multidisciplinary interventions because they combined assessment in more than one domain like the physical, cognitive and social domains together. In practice, being “eligible due to frailty” does not mean the same thing across studies, which is in line with recommendations elsewhere in the frailty literature for the need of having a shared way of defining frailty that also captures its severity, together with agreed outcome measures for quality of life, function, wellbeing, and participant experience [103].

Another term examined by this scoping review was the use of the homebound term. Although homebound status is a common reason for older adults to need care at home, it was seldom reported, even where study populations showed homebound characteristics. Homebound status was also subject to considerable variation in how consistently it was defined and reported. Only 14 studies clearly reported their population’s homebound status (11 identified as homebound and three as non-homebound). This low rate may be explained by the fact that the homebound literature still has no standardised measure, despite decades of study [7]. The remaining 50 studies (78.1%) were assessed using our PH/ARH framework. Of these, 27 were classified as “at risk of homebound”, six as “possibly homebound” and two as “non-homebound”. Nine studies included participants with a mix of severity levels and six could not be classified because of insufficient information. This graded picture is consistent with Ko and Noh’s (2021) own view that homebound status is better understood as a continuum and not as a binary condition [7]. The individual PH indicators, such as life-space confinement, ADL/IADL dependency and mobility limitation, are based on the Schirghuber and Schrems’ (2021) [9] concept analysis of homebound status, translating the broad homebound concept into criteria that could be applied consistently to study-level eligibility and baseline data. The nine studies with mixed classifications point to a further, practical implication. Within a single study, participants could be classified across more than one category (some as “possibly homebound,” others as “at risk of homebound” or “non-homebound”). This means a study’s overall population may contain a “hidden” subgroup of more severely confined participants, without this being recognised or analysed separately. Among the newly classified studies, at risk of homebound classifications were far more common than possibly homebound ones (27 vs. 6). This may suggest that most of this literature engages with populations before they reach confirmed home confinement, possibly capturing precursor factors such as frailty, multimorbidity, cognitive impairment, or social isolation, rather than established homebound status itself. If confirmed, these findings would support intervening during the at-risk stage, since this may be the stage much of the literature already engages with, even without recognising or labelling it as such. Even in studies that received a single classification (“at risk of homebound” or “possibly homebound”), applying the framework surfaced confinement characteristics that the studies had not identified. This reinforces the same concern that outcomes reported for a population as a whole may not reflect how well a service actually serves this more confined subgroup.

Risk of functional decline and frailty were the target groups most often classified as homebound or possibly homebound in this review. This is consistent with the literature describing a close and likely reciprocal relationship between these conditions. Herr et al. (2013) argued that homebound status itself should be considered an indicator of frailty, useful for identifying older adults likely to benefit from preventive intervention [3]. Hamazaki et al. (2016) [109] found that homebound status was a risk factor for later functional dependence in both men and women and that even men who were homebound but functionally intact at baseline remained at elevated risk of subsequent decline. A recent study found that while most homebound older adults are frail or pre-frail, a significant proportion are not and called for further research into this non-frail subgroup, whose status may be more reversible than frailty-related homebound status typically is [110]. However, studies that selected frail participants with a validated frailty tool were not more often classified as possibly homebound or at risk than those relying on clinical judgement. Frailty instruments, as used for eligibility, may therefore not specifically capture the difficulty in leaving the home that defines homebound status. The tools most commonly used in the included studies, such as the GFI and PRISMA-7, also do not include oral health, although oral frailty, a decline in functions such as chewing and swallowing [111], has been linked to broader functional decline, partly through leaving the home less often [112].

Homebound-relevant classifications were not limited to the frailty and functional-decline groups. They also appeared among psychosocially vulnerable populations, frequent service users and, less often, the general older population, groups not usually associated with physical confinement. Psychosocial determinants are known to contribute to homebound status [7]. In this review, the four studies in the psychosocially vulnerable group selected participants for reasons such as low income, living alone, unstable housing or living in an underserved area [40,48,52,99]. Only one of them described its population as homebound [52]. In the other three, the PH/ARH framework identified a risk of becoming homebound that had not been described. In these studies, different factors point to different routes towards home confinement. For instance, social isolation, which refers to a shortage of social contacts, was found to increase the risk of becoming homebound in community-dwelling older adults without cognitive impairment, and this risk was not mitigated even when participants lived with others [113]. Thus, living arrangement alone is not enough to understand who could be socially isolated. Older adults who live alone may warrant particular attention, but there is also evidence that older adults who live with others can still be socially disconnected, and this was even more common among those with limitations in daily activities [114]. Depressive symptoms also increase the likelihood of becoming homebound irrespective of cognitive status [113]. Depression and disability in daily activities appear to reinforce each other over time [115] and depressive symptoms are associated with a narrower life space, partly through walking difficulty and reduced autonomy outdoors [116]. One study in this review targeted two psychosocial factors, major depression and low income, in older adults who also had a disability [48]. The home-based intervention aimed to empower participants by teaching them problem-solving skills. Its population met several ARH indicators, including depressive symptoms, ADL or IADL limitations and multimorbidity, which indicate a population at risk of becoming homebound. Such populations may therefore be at a point where depression and declining function could reinforce each other and lead towards becoming homebound, if these risks are not addressed in time. Low income, the second psychosocial factor targeted in that study, is also more common among older adults who later become homebound [117]. Also, according to Steptoe and Zaninotto (2020), lower wealth is associated with faster decline in physical function in older adults, including slower walking and weaker grip [118]. In underserved areas [40], limited access to services is likely to matter most for those whose function is already declining. These findings suggest that the risk of becoming homebound is not limited to physical conditions, and that it should also be considered in psychosocially vulnerable groups, where it often remains unrecognised.

### 4.1. Implications for Further Research

Future research should look beyond frailty as the main explanation for homebound status, with particular attention to the non-frail homebound subgroup, whose status may be easier to reverse through targeted intervention. Longitudinal data support the reversibility of homebound status. In a cohort study of newly homebound Medicare beneficiaries, nearly half recovered within one year, though many became homebound again later and 65% had died within 6 years [10]. The reversibility of homebound status raises the hypothesis that a primary care screening tool could identify people at risk before or as they become homebound. Whether such a tool can be developed and validated, and whether earlier identification improves outcomes, are questions for future research. Depressive symptoms and social isolation, both associated with becoming homebound [113], may be relevant to such screening approaches. As living alone does not necessarily mean being socially isolated, it may serve as an early warning sign to check for isolation, rather than as a risk factor on its own.

Timely recognition of homebound risk would be consistent with the broader goal of healthy ageing, defined by the World Health Organization as maintaining the functional ability that enables wellbeing in older age [119]. Identifying individuals at risk earlier could also allow primary care to connect them proactively to home-based services, such as the models of home-based primary care and coordinated home visiting discussed throughout this review, instead of referring people to these services once functional decline or social isolation is already advanced.

### 4.2. Strengths

This review has several strengths. The search strategy combined five databases, grey literature and backward citation searching, with no restrictions on language or geographic region, and was updated in February 2026 to capture recently published studies. The review’s age threshold of 60, rather than the more commonly used 65, aligns with the World Health Organization’s broader international definition of older age and was intended to capture studies that might otherwise be excluded on age grounds alone. Lowering the age threshold to 60 did not mean the review ended up describing a younger, less relevant population. The studies found they still involved older participants, with mean ages ranging from 67 to 88.1 years. The review protocol was prospectively registered. Data was extracted by one reviewer and verified against the source publications by a second reviewer for all included studies, and the homebound classification was independently double coded in a 25% subsample. For the homebound status classifications, which required interpretive judgement, inter-rater agreement was formally assessed (κ = 0.949, 95% CI 0.88 to 1.00). This review also applied a classification framework, developed for this purpose, to describe homebound-consistent characteristics in study populations that did not use the term, a gap noted in the homebound literature [7]. While not yet an externally validated instrument, each indicator was grounded in established homebound literature, including the CMS Medicare criteria [8], the concept analysis by Schirghuber and Schrems [9] and the risk-factor taxonomy by Ko and Noh [7], and its classification threshold was empirically derived. The pattern of corroborating indicators found among studies that already explicitly reported homebound status was used to set the threshold, which was then applied to the separate subset of studies where homebound status was not reported, avoiding circularity between the studies used to derive the rule and those classified by it. In a sensitivity analysis, lowering the PH threshold to one indicator more than doubled the number of PH studies, while raising it to three indicators classified none. The threshold of two indicators was therefore retained as the most conservative one that still distinguished the PH from ARH studies. As such, the framework offers a starting point that could inform the development of a future primary care screening tool for identifying patients at risk of becoming homebound, rather than a finished instrument in itself.

### 4.3. Limitations

While the framework was developed from established literature and its threshold was empirically derived and tested through sensitivity analysis, it remains a framework developed by the review team specifically for this review and has not been externally validated, for example by comparing its classifications with direct assessment of whether participants are homebound. Eligibility criteria were verified by a second reviewer for all included studies, but agreement on this coding was not formally measured. As frailty, multimorbidity and cognitive impairment were also common eligibility criteria, the ARH classification partly reflects how study populations were selected and indicates a possible rather than confirmed risk of becoming homebound. Classification also depended on how much detail the study authors reported. Fifteen of the 50 studies (30.0%) assessed with the PH/ARH framework could not be placed into a single homebound-risk category, either because the study design produced multiple severity groups within the same population or because too little information was reported. Because the classifications are based on what studies reported and not on assessment of individual participants, they may partly reflect reporting practice and may underestimate homebound characteristics. This reflects a broader limitation that applies to all coded information in this review, not only homebound status. Where studies did not report sufficient detail, there is a possibility of misinterpretation or missed detail during coding, regardless of how carefully a framework or category is defined. In addition, interpretive coding was performed by one reviewer for most studies, with independent coding by a second reviewer limited to a 25% subsample.

The search strategy required terms related to needs assessment, reflecting the broader aims of the review protocol. Studies describing home-based care populations without reference to needs assessment may therefore have been missed. This risk was mitigated by including the broader term “health evaluation” in the search and by a thorough backward citation search of all studies assessed at full text, although the findings may not represent all home-based care studies. Although no language restrictions were applied, all included studies were published in English and studies published in other languages may be under-represented in the databases searched. Publication bias is also possible, as studies with null findings may be under-represented. Unpublished programmes may also have served different populations, particularly the most confined older adults, who are harder to recruit. Searching grey literature and a trial registry aimed to reduce this risk.

Each study was assigned to a single target population group, although some populations could plausibly fit more than one. Included studies also varied considerably in design, ranging from randomised controlled trials to quasi-experimental, implementation, and case studies. In keeping with JBI guidance for scoping reviews, risk of bias was not assessed, so all study designs contributed equally to this review’s synthesis, and no judgement is made about the methodological quality or certainty of the findings summarised here. Even basic demographic reporting varied widely. Age was described using at least five different formats across studies, which precluded combining this information into a single, consistent measure. Most included studies were conducted in the United States, the Netherlands, and Canada, so the patterns identified here may not generalise well to health systems with different models of primary care organisation, particularly in low- and middle-income countries.

## 5. Conclusions

This review found important variation in how cognitive status, functional dependency and frailty were defined and used across studies. Frailty, for example, was sometimes established using a validated tool and sometimes through clinical judgement alone. This makes comparison difficult, not only across studies, but also potentially within a single study, where different assessors may apply differing implicit thresholds. Homebound status itself was rarely reported by the original studies. This near absence of a consistently applied definition does not reflect a lack of conceptual work. The dimensions of homebound status have already been described in the literature but having a tool that operationalises these dimensions for clinical use is missing. Applying the classification framework developed for this review to the included studies suggested that homebound or at-risk of homebound characteristics are often present in study populations but are rarely described as such. This points to a reporting gap that limits how far findings can be applied to homebound older adults. This pattern was not confined to studies targeting frailty or functional decline. Homebound-relevant characteristics also appeared in several studies whose target populations were not defined by physical frailty or functional decline, including psychosocially vulnerable older adults. This suggests that screening for homebound risk may need to look beyond physical criteria. The at-risk classification reflects risk characteristics, not a prediction that participants will become homebound. Future research should examine whether a screening tool could help primary care identify patients at risk of becoming homebound before they lose the ability to attend. Such a tool, and any benefit of earlier identification, would first require external validation and prospective evaluation.

## Figures and Tables

**Table 1:** Summary of inclusion and exclusion criteria applied to citations identified through the literature search.

	Inclusion Criteria	Exclusion Criteria
Population	Age ≥ 60 years old Both sexes Living in the community (non-institutional settings)	Age < 60 years old Residing in nursing homes or assisted living facilities, senior housing, long-term/chronic care facilities, prisons, detention facilities, asylum centres
Intervention characteristics	Implemented by primary healthcare professionals, community healthcare professionals, or case managers Delivered at home with more than one visit Covers: provision of treatment of chronic conditions, disability management, urgent care, preventive care, post-discharge care, care coordination, and environmental assessments, mental health support, palliative care	One-time visits or evaluations Home visits for emergency care Post-operative home visits for follow-up within 3 months from discharge
Context/ Setting	Studies focusing on the delivery and/or development of primary care or community care services at home to improve health of population	Studies focusing on services intended to be implemented in chronic care facilities, healthcare facilities, practice or clinic, nursing homes, assisted living homes, senior housing and prisons, detention facilities, asylum centres
Outcomes	Studies aimed at the improvement of individuals’ health and wellbeing (e.g., enhance self-management, disease prevention, chronic disease management, medication adherence, lifestyle modifications, and patient education)	Exclusive endpoint: satisfaction and/or change in perceived burden among informal caregivers Exclusive endpoint: effectiveness of telemedicine applications or e-health tools Exclusively discusses cost-effectiveness, survival, hospitalisation and readmission rates
Study characteristics (type of evidence)	Observational studies (retrospective and prospective) Interventional studies (randomised and non-randomised interventional trials) Dissertations and theses Qualitative and narrative studies All languages and geographies From database inception	Literature reviews Editorials, opinion papers News and websites Letters, conference abstracts/proceedings, government and/or professional organisation reports Study protocols

**Table 2:** General characteristics of the included studies (*n* = 64).

Characteristics	Category	Number of Studies	%	Citations
Country	USA	16	25.0	[39,40,41,42,43,44,45,46,47,48,49,50,51,52,53,54]
	Netherlands	9	14.1	[55,56,57,58,59,60,61,62,63]
	Canada	9	14.1	[36,64,65,66,67,68,69,70,71]
	Sweden	4	6.3	[72,73,74,75]
	Australia	3	4.7	[76,77,78]
	Germany	3	4.7	[37,79,80]
	Switzerland	3	4.7	[81,82,83]
	Norway	2	3.1	[84,85]
	Japan	2	3.1	[86,87]
	Hong Kong	2	3.1	[88,89]
	United Kingdom	2	3.1	[38,90]
	Finland	2	3.1	[91,92]
	France	2	3.1	[93,94]
	Ireland (Northern Ireland and Republic of Ireland cross border study)	1	1.6	[95]
	Spain	1	1.6	[96]
	Singapore	1	1.6	[97]
	Taiwan	1	1.6	[98]
	Turkey	1	1.6	[99]
Publication year	1990–1999	4	6.3	[53,71,87,90]
	2000–2009	9	14.1	[38,39,52,63,70,78,83,86,94]
	2010–2019	25	39.1	[36,37,47,48,49,50,51,54,56,57,58,59,60,61,62,69,72,73,74,75,77,81,82,85,92]
	2020–2026	26	40.6	[40,41,42,43,44,45,46,55,64,65,66,67,68,76,79,80,84,88,89,91,93,95,96,97,98,99]
Design	Randomised controlled trials	32	50.0	[38,44,48,50,53,57,59,61,62,63,64,67,68,69,70,72,73,74,75,76,77,81,82,83,85,86,88,89,90,92,95,98]
	Quasi-experimental/Pilot	17	26.6	[37,40,41,42,43,45,46,49,55,60,78,80,84,91,96,97,99]
	Implementation studies	12	18.8	[36,47,51,54,56,58,65,66,71,87,93,94]
	Case studies	2	3.1	[39,52]
	Secondary analysis	1	1.6	[79]
Sex distribution	Female participants ≥ 50%	49	76.6	[36,37,40,43,44,47,49,50,51,53,54,55,56,57,59,60,62,63,64,65,66,67,69,70,71,72,73,74,75,76,77,78,79,80,81,82,83,84,85,86,88,89,90,91,92,94,97,98,99]
	Female < 50% of participants	8	12.5	[39,42,45,52,61,68,95,96]
	Not reported	7	10.9	[38,41,46,48,58,87,93]
Age of participants	Mean age	45	70.3	[37,39,40,42,43,44,45,48,49,50,53,54,55,56,59,60,61,62,63,64,65,69,70,71,72,73,76,77,78,79,81,82,83,84,85,86,87,89,91,92,94,95,96,97,98]
	Median age	3	4.7	[66,75,88]
	Age categories	6	9.4	[36,67,68,80,90,99]
	Age threshold	5	7.8	[38,47,51,74,93]
	Not reported	5	7.8	[41,46,52,57,58]
Focus/rationale of intervention	Care coordination/integration	24	37.5	[39,40,44,45,51,52,54,55,64,68,71,72,73,74,75,81,84,87,88,89,94,96,97,98]
	Preventive and early-detection	15	23.4	[53,56,58,60,62,63,69,70,80,82,83,86,90,93,95]
	Disease/symptom management	10	15.6	[37,38,41,42,43,50,66,67,79,92]
	Functional independence/reablement	7	10.9	[47,59,76,77,78,85,91]
	Psychosocial support	4	6.3	[48,57,61,99]
	Palliative/end-of-life care	4	6.3	[36,46,49,65]

**Table 3:** Target population characteristics (*n* = 64).

Target Characteristic	Number of Studies (%)	Studies
Frail or at risk of frailty patient	20 (31.3%)	[39,50,51,55,56,57,58,59,62,65,70,72,73,74,75,81,84,86,87,94]
Patients with risk of functional decline	11 (17.2%)	[44,47,54,63,71,78,83,85,91,93,95]
Patient with multimorbidity or complex chronic conditions	10 (15.6%)	[36,46,49,61,66,67,68,96,97,98]
General older population	8 (12.5%)	[53,60,69,80,82,88,89,90]
Dementia or cognitive impairment	6 (9.4%)	[41,42,43,76,77,79]
Psychosocial vulnerable populations	4 (6.3%)	[40,48,52,99]
Medication related risks	3 (4.7%)	[37,38,92]
Frequent service users	2 (3.1%)	[45,64]

**Table 4:** Eligibility criteria.

Criterion	Number of Studies (%)	Studies
Cognitive status		
Cognitive status used as a marker of consent capacity, literacy or broader frailty assessment	15	[38,45,46,50,55,65,80,81,82,83,84,85,94,95,96]
Cognitive status used to exclude cognitive impairment	11	[47,53,67,68,69,73,75,78,88,89,91]
Cognitive status as an inclusion criterion for patients living with dementia	6	[41,42,43,76,77,79]
Total	32 (50.0%)	
Functional Status		
Functional status to determine dependency as an inclusion criterion	25	[37,41,44,46,47,48,49,50,51,52,54,63,65,70,71,73,74,75,77,78,83,84,85,91,94]
Functional status to assess independence as an inclusion criterion	2	[53,69]
Total	27 (42.2%)	
Frailty		
Presence of frailty as an inclusion criterion	22	[39,49,50,51,55,56,57,58,59,62,65,70,72,73,74,75,81,84,86,87,94,95]
Absence of frailty as an inclusion criterion	1	[89]
Total	23 (35.9%)	
Multimorbidity	12 (18.8%)	[37,39,46,51,67,68,72,74,83,84,95,96]
Polypharmacy	7 (10.9%)	[38,50,56,58,72,83,95]

Note: Studies combined more than one criterion.

**Table 5:** Frailty eligibility criteria and their comparison with the target populations of each study (*n* = 23).

Category	Study	Validated Tool/Scale/Criteria Combination Used
Studies targeting frail patients using a named or validated frailty-related tool/index (alone or in combination with professional judgement) (*n* = 7)	Di Pollina et al. (2017) [81]	RAI-HC (Resident Assessment Instrument—Home Care edition). Frailty inclusion required at least one of four alarms/risk factors of the RAI-HC and if one was present, frailty was further quantified using the CA+.
	Bleijenberg et al. (2016) [56]	Frailty calculated from GP electronic medical record data using a frailty index.
	Muntinga et al. (2015) [58]	PRISMA-7 ≥ 3, combined with a GP’s composite definition of frailty and a polypharmacy criterion.
	Metzelthin et al. (2013) [59]	GFI ≥ 5
	van Hout et al. (2010) [62]	COOP-WONCA (worst quartile on ≥2 of 6 domains)
	Vedel et al. (2009) [94]	CA+ tool score ≥ 6/9 in combination with primary care physician judgement.
	Ritchie et al. (2002) [39]	PRA-Plus score ≥ 0.35
Studies targeting frail patients using clinical judgement or a combination of unstructured multidimensional factors (*n* = 13)	Hoedemakers et al. (2022) [55]	GP/nurse practitioner clinical/case-finding judgement.
	Islam et al. (2021) [84]	Frail patient with multimorbidity (≥2 conditions) starting or extending municipal home care services.
	Cox et al. (2021) [65]	Inability to access usual care due to physical or cognitive frailty and the presence of a high degree of frailty based on dependency on others for basic activities of daily living.
	Fristedt et al. (2019) [72]	Multimorbidity (>3 conditions) combined with polypharmacy (≥6 medications) and hospitalisation frequency (≥3 hospital stays during the last 6 months).
	Taube et al. (2018) [73]	Functional dependency and repeated contacts with the healthcare services.
	Dorresteijn et al. (2016) [57]	Self-reported fair or poor perceived health, at least some concerns about falls and activity avoidance due to concerns about falling.
	Eklund et al. (2013) [74]	Age combined with at least 1 ADL dependency and ≥1 chronic disease. Use of eight frailty indicators (weakness, fatigue, weight loss, low physical activity, poor balance, low gait speed, visual impairment, cognitive impairment) following enrolment.
	Marek et al. (2013) [50]	Medication-management difficulties and/or impaired cognitive functioning.
	Allen et al. (2011) [51]	Functional dependency and ≥1 chronic condition.
	Kristensson et al. (2010) [75]	Functional dependency and ≥2 hospital admissions/≥4 GP contacts during the previous year.
	Kono et al. (2009) [86]	Lowest 2 support levels of the Long-Term Care Insurance (LTCI) (administrative classification).
	Dalby et al. (2000) [70]	Self-reported multidimensional questionnaire about functionality, social activities, perceived health and use of services/admission to hospital.
	Murashima et al. (1998) [87]	Clinical nurse judgement
Studies using validated frailty scales/tools as eligibility criteria, targeting patients not characterised as frail (*n* = 3)	Brazil et al. (2022) [95]	PRISMA-7 > 2
	Wong et al. (2020) [89]	Clinical Frailty Scale (CFS) < 5 (frailty used as an exclusion criterion)
	Chen et al. (2015) [49]	ERA score > 16

**Table 6:** Classification of homebound status across included studies that reported homebound status (*n* = 14).

Homebound Criteria	Study	Description
Studies using a formal homebound definition (*n* = 4)	Federman et al., 2023 [44]	Criteria approximated the Medicare homebound definition (≥1 hospitalisation in prior 12 months, need for assistance with ≥2 ADLs, leaving home ≤ 2 times/week).
	Mendez et al., 2020 [46]	Individuals that demonstrated taxing effort or required assistance to leave home, (consistent with the Medicare definition).
	Reckrey et al., 2016 [54]	Medicare homebound definition (individuals able to leave home only with great difficulty and for short duration or infrequently).
	Chen et al., 2015 [49]	Medicare and Medicaid definition of homebound.
Studies combining functional and clinical dimensions in the eligibility criteria to describe home confinement or homebound condition (*n* = 4)	Cox et al., 2021 [65]	Homebound status described as “inability to access usual primary care due to physical or cognitive frailty” and “a high degree of frailty based on dependency on others for basic ADLs”.
	Tuntland et al., 2015 [85]	The control group was described as homebound and the intervention group was recruited through the same referral pathway, on the basis of functional decline and need for home-based services.
	Mai and Eng, 2007 [52]	Poor functioning affecting motor ability/ambulation or impaired ADLs (e.g., difficulty toileting or walking without an assistive device).
	Clarfield and Bergman, 1991 [71]	Homebound was described as a person needing care at home.
Homebound status as a baseline characteristic of participants and other relevant description in the study (*n* = 3)	Ritchie et al., 2025 [41]	It was described that the intervention was adjusted to the needs of homebound people living with dementia. Participants had a dementia diagnosis, an available caregiver, need for assistance with at least 2 IADLs or 1 ADL and caregiver distress judged by the primary clinician.
	Nadin et al., 2018 [36]	A case example in the baseline description reports a client as “mostly homebound”; needing palliative services at home. All participants suffered from advanced chronic or terminal illness and requested palliative care at home.
	Murashima et al., 1998 [87]	Baseline dependency table reports participants as house-bound, chair-bound, or bed-bound. All participants, even those mentioned as almost independent, were nursing care services recipients.
Non-homebound (*n* = 3)	Wong et al., 2020 [89]	Excluded bed-bound, cognitive impaired and frail participants.
	Godwin et al., 2016 [69]	Target population is described as independent living, functioning well cognitively.
	Kono et al., 2009 [86]	Population is described as ambulatory, generally independent in most ADLs and not seriously cognitively impaired.

**Table 7:** Indicator definitions and decision rules applied to classify home-confinement risk among studies with no direct indication of homebound status.

Indicator	Definition
Possibly Homebound (PH) indicators	
PH1	Dependency on another person to leave the home (e.g., cannot go out unattended).
PH2	Dependence on IADL or ADL that potentially limits mobility. (Note: all ADL/IADL instruments reported in each study (e.g., Katz-15, B-ADL, Barthel Index) were assessed, not a single scale in isolation. If no instrument was used, a narrative description of ADL/IADL dependence is also acceptable, if it names specific tasks (e.g., managing medications, housekeeping) rather than using general statements of disability or need for assistance.
PH3	Reported limitations in walking or moving outdoors (e.g., can only walk short distances independently, has difficulty walking and climbing stairs, moving around in the house is possible with a stick, a walking aid, or by holding on to furniture). (Note: the limitation must be reported in the study and not inferred from a PH2 instrument score alone. Also, the same information cannot be coded as PH2 and PH3.)
PH4	Already receiving home-based health or personal care services (e.g., home nursing, home physiotherapy, home palliative care, home-based primary care), indicating increased care need and possible difficulty in attending a practice or clinic. Only services received before and independently of the study intervention were coded. Services limited to home-delivered meals or domestic help were not counted.
PH5	Life-space restriction or “confined to home”.
PH6	Environmental factors limiting transport combined with functional impairment, directly linked in the study as jointly restricting community access (e.g., “stairs at the home entrance combined with the reduced mobility prevent them from leaving unassisted”).
At Risk of Homebound (ARH) indicators	
ARH1	Frailty, sarcopenia, falls or fear of falling, gait impairment, weakness.
ARH2	Multimorbidity (≥2 conditions) (Note: in studies that did not provide a count of comorbidities, other indicators consistent with multimorbidity (e.g., polypharmacy/number of regular medications, Charlson Comorbidity Index category) were considered as supporting evidence.)
ARH3	ADL/IADL limitations not yet restricting community mobility (Note: all ADL/IADL instruments reported in each study (e.g., Katz-15, B-ADL, Barthel Index) were assessed, not a single scale in isolation.)
ARH4	Cognitive impairment, without documented confinement at home.
ARH5	Depressive symptoms, anxiety, loneliness, or low sense of coherence, without confirmed activity restriction.
ARH6	Social determinants: social isolation, inadequate caregiving or social support needs, due to low income, loss of social role, or living alone, without evidence of home confinement.
ARH7	Environmental factors: stairs at home entrance, heavy doors, poor neighbourhood walkability, lack of transport access, or rural location, without confirmed home confinement statement linking them to functional impairment as jointly restricting access. (Note: If the study reports that the combination of environmental factor and functional impairment restrict access, it is coded as PH6 instead.)
Decision rules	
PH	At least two PH indicators, regardless of the number of ARH indicators.
ARH	One PH indicator, or no PH indicator and at least one ARH indicator.
Not homebound	No PH and no ARH indicator, with participant information sufficient to show their absence (decision made by reviewing the study).
Mixed populations	The study population included subgroups that would fall into different categories (PH, ARH or not homebound), either because the eligibility criteria created separate entry routes or strata or because the reported sample included participants both with and without functional dependency. No single category was assigned.
Insufficient information	Participant characteristics were not reported in enough detail to assess the indicators. No category was assigned.

Notes: The PH threshold of two indicators reflects the pattern observed in the studies that defined or described homebound status, which usually combined at least two corroborating elements (Appendix C). In a sensitivity analysis, lowering the PH threshold to one indicator more than doubled the number of PH studies (from 6 to 13), while raising it to three classified none, as no study met more than two PH indicators (Appendix C).

**Table 8:** Classification of home-confinement risk among studies with no direct indication of homebound status (*n* = 35).

Study	Indicators Met	Target Group
Possibly Homebound (PH) (*n* = 6)		
Islam et al., 2021 [84]	PH2, PH4, ARH1, ARH2	Frail or at risk of frailty patient
Toivo et al., 2019 [92]	PH3, PH4, ARH2, ARH4, ARH6	Medication related risks
Fristedt et al., 2019 [72]	PH1, PH4, ARH1, ARH2, ARH3	Frail or at risk of frailty patient
Di Pollina et al., 2017 [81]	PH2, PH4, ARH1	Frail or at risk of frailty patient
Fiss et al., 2010 [37]	PH4, PH5, ARH2	Medication related risks
Vedel et al., 2009 [94]	PH1, PH2, ARH1, ARH2, ARH4, ARH6	Frail or at risk of frailty patient
At Risk of Homebound (ARH) (*n* = 27)		
Teerikangas et al., 2026 [91]	PH3, ARH1, ARH3	Patients with risk of functional decline
Posey et al., 2026 [40]	ARH1, ARH5	Psychosocial vulnerable populations
Ciemins et al., 2025 [42]	ARH2, ARH4	Dementia or cognitive impairment
Aïdoud et al., 2025 [93]	ARH1	Patients with risk of functional decline
Gitlin et al., 2024 [43]	ARH1, ARH4	Dementia or cognitive impairment
Arabacı et al., 2024 [99]	PH3, ARH1, ARH6	Psychosocial vulnerable populations
Michalowsky et al., 2024 [79]	ARH2, ARH3, ARH4	Dementia or cognitive impairment
Blotenberg et al., 2023 [80]	ARH2, ARH7	General older population
Mangin et al., 2023 [64]	ARH3	Frequent service users
Hoedemakers et al., 2022 [55]	PH2, ARH1, ARH2	Frail or at risk of frailty patient
Brazil et al., 2022 [95]	ARH1, ARH2	Patients with risk of functional decline
Ohuabunwa et al., 2021 [45]	ARH2, ARH6	Frequent service users
Miklavcic et al., 2020 [67]	ARH2	Patient with multimorbidity or complex chronic conditions
Fisher et al., 2020 [68]	PH4, ARH2, ARH5	Patient with multimorbidity or complex chronic conditions
Mateo-Abad et al., 2020 [96]	ARH1, ARH2	Patient with multimorbidity or complex chronic conditions
Bleijenberg et al., 2016 [56]	ARH1, ARH2	Frail or at risk of frailty patient
Alexopoulos et al., 2016 [48]	ARH2, ARH3, ARH5	Psychosocial vulnerable populations
Dorresteijn et al., 2016 [57]	ARH1, ARH3, ARH5	Frail or at risk of frailty patient
Szanton et al., 2016 [47]	PH2, ARH6	Patients with risk of functional decline
Marek et al., 2013 [50]	ARH1, ARH2, ARH3, ARH4	Frail or at risk of frailty patient
Eklund et al., 2013 [74]	PH2, ARH1, ARH2	Frail or at risk of frailty patient
Jonkers et al., 2012 [61]	ARH2, ARH5	Patient with multimorbidity or complex chronic conditions
van Hout et al., 2010 [62]	ARH1, ARH2	Frail or at risk of frailty patient
Lewin and Vandermeulen, 2009 [78]	PH4, ARH1, ARH3	Patients with risk of functional decline
Lenaghan et al., 2007 [38]	ARH2, ARH6	Medication related risks
van Haastregt et al., 2000 [63]	ARH1, ARH2	Patients with risk of functional decline
Dalby et al., 2000 [70]	ARH1, ARH2	Frail or at risk of frailty patient
Not Homebound (*n* = 2)		
Bayuo et al., 2025 [88]	None	General older population
Raube et al., 1992 [53]	None	General older population

Notes: Indicator definitions and decision rules are presented in Table 7, with detailed coding guidance in Appendix C. PH: at least two PH indicators. ARH: one PH indicator, or no PH indicator and at least one ARH indicator. Studies with a mixed population (*n* = 9) or insufficient information (*n* = 6) are not presented in this table, which reports studies that fit into a single classification.

## Data Availability

The original data presented in the study are available at https://doi.org/10.6084/m9.figshare.34031538 (accessed on 30 September 2026).

## Supplementary Materials

The following supporting information can be downloaded at: https://www.mdpi.com/article/10.3390/healthcare14213670/s1, Supplementary File: PRISMA-ScR checklist.

## Abbreviations

The following abbreviations are used in this manuscript:

ACP	Anticipatory Care Planning
ADL	Activities of Daily Living
AIMS	Age-Friendly Integrative Mobile Services
ARH	At Risk of Homebound
CA+	Contact Assessment plus
CARE model	Coordination and Advocacy for Rural Elders
CCFE	Care Chain Frail Elderly
C-HSPP	Community-based health–social partnership programme
CFS	Clinical Frailty Scale
COOP-WONCA	Dartmouth COOP Functional Health Assessment Charts by the World Organization of Family Doctors
ERA	Elder Risk Assessment
GFI	Groningen Frailty Indicator
GP	General Practitioner
HCOACDM	High-Need Community-Dwelling Older Adults Care Delivery Model
IADL	Instrumental Activities of Daily Living
LTCI	Long-Term Care Insurance
MMSE	Mini Mental State Examination
MoCA	Montreal Cognitive Assessment
NPI-Q	Neuropsychiatric Inventory-Questionnaire
OASIS	Outcome and Assessment Information Set
OSF	Open Science Framework
PCC framework	Population, concept and context framework
PH	Possibly Homebound
PRA-Plus score	Probability of Repeated Admission Score
PRISMA-ScR	PRISMA Extension for Scoping Reviews
RAI-HC	Resident Assessment Instrument—Home Care edition
SPMSQ	Short Portable Mental Status Questionnaire
U-PROFIT	Utrecht PROactive Frailty Intervention Trial

## Appendix A. Search Strategy Adapted to Databases

**Database**	**Search Strategy**
Pubmed	(((((((((aged[MeSH Terms]) OR (aged[Title/Abstract])) OR (aging[MeSH Terms])) OR (“elder*”[Title/Abstract])) OR (old[Title/Abstract])) OR (senior[Title/Abstract])) OR (geriatric[Title/Abstract])) AND ((((((((((((Family Practice[MeSH Terms]) OR (“family medicine”[Title/Abstract])) OR (General Practice[MeSH Terms])) OR (General Practitioners[MeSH Terms])) OR (Physicians, Primary Care[MeSH Terms])) OR (Primary Health Care[MeSH Terms])) OR (Physician assistant[MeSH Terms])) OR (Primary Care Nursing[MeSH Terms])) OR (Community Health Nursing[MeSH Terms])) OR (“health visitors”[Title/Abstract])) OR (“practice nurs*”[Title/Abstract])) OR (case management[MeSH Terms])) OR (“case managers”[Title/Abstract]))) AND (((Needs Assessment[MeSH Terms]) OR (“health needs assessment”[Title/Abstract])) OR (“health evaluation”[Title/Abstract])) AND ((((((((home care services[MeSH Terms]) OR (“home visit*”[Title/Abstract])) OR (home[Title/Abstract])) OR (“home-based”[Title/Abstract])) OR (“at-home”[Title/Abstract])) OR (house calls[MeSH Terms])) OR (homebound[Title/Abstract])) OR (homebound persons[MeSH Terms]))
Cochrane Central Register of Controlled Trials (CENTRAL)	(MeSH descriptor: [Aged] explode all trees OR MeSH descriptor: [Aged] in all MeSH products OR (aged):ti,ab,kw OR (elder*):ti,ab,kw OR (old):ti,ab,kw OR (senior):ti,ab,kw OR (geriatric):ti,ab,kw) AND (MeSH descriptor: [Family Practice] explode all trees OR MeSH descriptor: [General Practice] explode all trees OR MeSH descriptor: [General Practitioners] explode all trees OR MeSH descriptor: [Physicians, Primary Care] explode all trees OR MeSH descriptor: [Primary Health Care] explode all trees OR MeSH descriptor: [Physician assistant] explode all trees OR MeSH descriptor: [Primary Care Nursing] explode all trees OR MeSH descriptor: [Community Health Nursing] explode all trees OR (“family medicine”):ti,ab,kw OR (practice NEXT nurs*):ti,ab,kw OR (“health visitors”):ti,ab,kw OR MeSH descriptor: [Case Management] in all MeSH products) AND (MeSH descriptor: [Needs Assessment] explode all trees OR (“health needs assessment”):ti,ab,kw OR (“health evaluation”):ti,ab,kw) AND (MeSH descriptor: [Home Care Services] explode all trees OR MeSH descriptor: [House Calls] explode all trees OR MeSH descriptor: [Homebound Persons] explode all trees OR (home NEXT visit*):ti,ab,kw OR (home):ti,ab,kw OR (“home-based”):ti,ab,kw OR (“at-home”):ti,ab,kw OR (homebound):ti,ab,kw)
Scopus	TITLE-ABS-KEY ( aged OR aging OR elder* OR old OR senior OR geriatric ) AND TITLE-ABS-KEY(“Family Practice” OR “family medicine” OR “General Practi*” OR “Primary Care Physicians” OR “Primary Health Care” OR “Physician assistant” OR “Primary Care Nursing” OR “Community Health Nursing” OR “health visitors” OR “practice nurs*” OR “case management” OR “case managers”) AND TITLE-ABS-KEY(“Needs Assessment” OR “health needs assessment” OR “health evaluation”) AND TITLE-ABS-KEY(“home care services” OR “home visit*” OR home OR “home-based” OR “at-home” OR “house calls” OR homebound OR “homebound persons”)
ProQuest	(noft(aged) OR noft(geriatric) OR noft(aging) OR noft(elder) OR noft(senior)) AND (noft((general practitioner)) OR noft((primary care physicians)) OR noft((physician assistant)) OR noft((primary care nurs*)) OR noft((Community Health Nurs*)) OR noft((health visitors)) OR noft(case manager) OR noft((case management))) AND (noft(home care services) OR noft((home visit*)) OR noft(homebound)) AND (noft(assessment needs) OR noft((health needs assessment)) OR noft(health evaluation)))
ClinicalTrials.gov	Homebound OR Elderly/ Community Health Worker OR Nursing care OR Primary care OR Physician assistant OR Case management OR case-manager health needs assessment OR Home Care

## Appendix B. Title/Abstract Screening Tool

**Title/Abstract Screening Tool**
Title and Abstract Screening 1. Does the title or abstract NOT indicate that a review, systematic review or meta-analysis was conducted? a. Yes: continue screening b. No: stop screening 2. Does the title or abstract indicate that this is NOT a study protocol, an editorial, opinion paper, letter, conference abstracts/proceedings, government and/or professional organisation report or news/website? a. Yes: continue screening b. No: stop screening Abstract Screening 3. Does the abstract indicate that a sample 60 years of age or older was the subject of the study? a. Yes or Unsure/Unclear: continue screening b. No: stop screening 4. Does the abstract indicate that community-dwelling subjects participated in the study? a. Yes or Unsure/Unclear: continue screening -Key words: home, non-institutional setting, private houses/residencies or apartments b. No: stop screening -Key words: nursing homes, assisted living facilities, senior housing, long-term/chronic care facilities, prisons, detention facilities, asylum centres 5. Does the abstract indicate that home was the site of service delivery in the study? a. Yes or Unsure/Unclear: continue screening b. No: stop screening 6. Does the abstract indicate primary healthcare services being delivered? a. Yes or Unsure/Unclear: continue screening -Key words: primary care, community-based care, integrated primary care, community oriented primary care -Key terms: preventive care, provision of treatment of chronic conditions, disability management, urgent care, post-discharge care, care coordination, and environmental assessments, mental health support, palliative care b. No: stop screening 7. Does the abstract NOT indicate healthcare services being used to address emergency cases? a. Yes or Unsure/Unclear: continue screening -Key point: If the study refers to urgent care/evaluation/assessment, then continue screening b. No: stop screening -Key terms: emergency care, emergency evaluation, life-threatening assessment/evaluation, immediate provision of assessment/care 8. Does the abstract NOT indicate healthcare services being used to address immediate post-operative care? a. Yes: continue screening b. No: stop screening -Key words: post-surgery -Key terms: late post-operative period or when it is indicated that care related to the surgery is provided within three months of surgery Decision: Should this article be included? a. Yes, all 8 screening questions answered Yes or Unclear b. No, at least one answers “No”
Note: The title/abstract screening tool has objective items, single-component questions (yes/no/unsure answers) [32]. The tool was piloted by the two reviewers on a random selection of citations and refined according to reviewers’ comments.

## Appendix C. Extraction Form and Coding Manual

**1. Study Characteristics**	
Items	Description/codes
Study title	Full title of the article
Author(s)	All listed authors
Year of publication/Journal	Year the study was published and journal
Country	Country in which the study was conducted
Rural setting	Whether the study was conducted exclusively in a rural area (yes or no)
Study and Intervention aim/objective	Classified according to the focus and rationale of the delivered intervention and what the programme itself was designed to achieve (e.g., disease management, prevention, rehabilitation) and through what mechanism (e.g., care management).
Study design	Classification of studies according to methodological design and purpose. 1. Randomised controlled studies: Studies in which participants/clusters were randomly allocated to intervention or control groups, regardless of blinding status. Includes individual RCTs, cluster RCTs, three-arm RCTs, non-blinded RCTs, feasibility cluster RCTs, and RCT design papers. 2. Quasi-experimental/pilot: Studies that evaluated an intervention without random allocation of participants to a group or pilot/ feasibility studies with the primary aim of preliminary testing. Pre–post designs, non-randomised controlled studies, retrospective cohort studies with matched controls and pilot or feasibility studies using non-randomised or descriptive designs were included. 3. Implementation studies: Studies whose primary aim was to evaluate the process and the delivery of an intervention under real conditions rather than its effectiveness. 4. Case study: Studies or reports describing the design or early implementation of an intervention without comparative design. 5. Secondary analysis: Post hoc analysis of data from a previously conducted trial or study.
Study sample and demographics	Sample size and characteristics of participants (e.g., age range, sex distribution, how the age rage is reported).
Involved professionals	Healthcare professionals involved in the delivery of the intervention/ service. Presentation of the different specialties involved in the interventions by frequency and classified according to the overall team structure (e.g., presence of a leading specialty).
Intervention characteristics	For example, duration and frequency of visits, follow-up
2. Target Population Characteristics and Eligibility Criteria	
Items	Description/codes
Target population of the study	Main characteristics of the target population (e.g., community-dwelling older adults living with dementia). Report if informal caregiver/ family member was recruited.
Inclusion criteria	Reported inclusion criteria.
Note: Exclusion criteria served to understand in more depth and clarify the characteristics of the included population and the setting of the study.	
3. Use of “Homebound” Status as Eligibility Criterion	
Items	Description/codes
Is “homebound”/“confined at home” used as an eligibility criterion?	This question is a Yes/ No question. Answer “yes” in case the study explicitly reports the term “homebound”, “housebound”, or “home-limited”, “confined at home”.
If the answer is “yes” highlight if a formal definition is reported in the description of the study population or the eligibility criteria or present how authors describe the homebound patient: Some definitions provided in literature are: 1. According to Medicare Home Health Criteria [8], “a person is considered homebound when illness or injury means that leaving home requires supportive devices, special transportation, or assistance from another person, or when leaving home is medically contraindicated, and when the person normally cannot leave home and doing so requires a considerable and taxing effort. Infrequent or short absences, including for health care, do not necessarily negate homebound status.” 2. According to Schirghuber and Schrems (2021) concept analysis, homebound is an enduring condition in which “the life-space is reduced to the home, but moving around in the home is possible. Six defining attributes are present: (1) need for help in ADL/IADL and leaving the life-space, (2) powerlessness, (3) life-space confinement, (4) mobility limitation, (5) endurance of the condition, and (6) weakness.” [9] 3. Uses a classification that quantifies the frequency of leaving home and the need for assistance [4]. Homebound is classified as “(1) completely homebound: never left home in the past month, (2) mostly homebound: rarely left home (once a week or less), (3) semi-homebound: left home 2+ days/week but with difficulty, assistance, or never alone.” 4. According to Ko and Noh (2021) scoping review, there is a variety of definitions [7]. Some definitions describe the individual as “confined to home” or “remaining inside the home” due to medical or mobility reasons. In some studies frequency is part of the definition and participants are homebound when going out “once a week or less”, “rarely”, or “never” and unable to leave home independently without assistance from another person. In other studies, a combination of multiple factors is taken into consideration to determine the status of homebound. These are the time spent at home, the frequency of going out, frequency of social contact outside home, and need for help. 5. Other national policies.	
When the answer is “no”, but participant still exhibit characteristics that could imply home confinement, the eligibility criteria and baseline characteristics of the participants need to be checked against a pre-set coding framework, to identify if the participants were “possibly homebound”, if they presented risk factors for developing a homebound status “at risk of homebound” or if they presented no homebound characteristics. Coding framework Each item in the coding framework was drawn from studies describing the concept of homeboundness and/or a combination of existing definitions [4,7,8,9]. Possibly Homebound (PH) This code is applied when the study provides a strong description of home confinement through functional, clinical or service-use indicators. Possibly Homebound indicators (PH): ≥2PH (regardless of how many ARH) 1. Dependency on another person to leave the home (e.g., cannot go out unattended) [7,8]. 2. Dependence in IADL or ADL that potentially limits mobility. (Note: all ADL/IADL instruments reported in each study (e.g., Katz-15, B-ADL, Barthel Index) were assessed, not a single scale in isolation. If no instrument was used, a narrative description of ADL/IADL dependence is also acceptable, if it names specific tasks (e.g., managing medications, housekeeping) rather than using general statements of disability or need for assistance [9]. 3. Reported limitations in walking or moving outdoors (e.g., can only walk short distances independently, has difficulty walking and climbing stairs, moving around in the house is possible with a stick, a walking aid, or by holding on to furniture) [9]. 4. Already receiving home-based health or personal care services (e.g., home nursing, home physiotherapy, home palliative care, home-based primary care. Services limited to home-delivered meals or domestic help were not counted. This is taken as an indicator for increased need for care and possible difficulty in a practice or clinic [11]. Only services received before and independently of the study intervention were coded. 5. Life-space restriction or “confined to home” mentioned in the study [7,9]. 6. Environmental factors limiting transport combined with functional impairment, directly linked in the study as jointly restricting community access (e.g., “stairs at the home entrance combined with the reduced mobility prevent them from leaving unassisted”) [120]. At Risk of Homebound (ARH) At Risk of Homebound (ARH) indicators: PH = 0, ARH ≥ 1 or PH = 1, ARH = 0 or PH = 1, ARH ≥ 1 This code is applied when one or more major determinants of homebound status are reported without evidence of actual home confinement. Participants are vulnerable. The risk factors are grounded in Ko & Noh’s (2021) ecological three-domain model (individual, social, environmental), McManus et al.’s (2025) risk factor taxonomy, and studies by Yang et al., 2021 and Choi et al., 2025 [6,7,113,120]. 1. Frailty, sarcopenia, falls or fear of falling, gait impairment, weakness, with no documented home confinement: (frailty/sarcopenia: [6,7,121]—falls/fear of falling: [7,122]—gait impairment/weakness: [7,9]); 2. Multimorbidity (≥ 2 conditions) (AMS, 2018) (note: in studies that did not provide a count of comorbidities, other indicators consistent with multimorbidity (e.g., polypharmacy/number of regular medications, Charlson Comorbidity Index category) were considered as supporting evidence) [123]; 3. ADL/IADL limitations not yet restricting community mobility [7] (note: all ADL/IADL instruments reported in each study (e.g., Katz-15, B-ADL, Barthel Index) were assessed, not a single scale in isolation); 4. Cognitive impairment, without documented restriction to home [7,113]; 5. Depressive symptoms, anxiety, loneliness, or low sense of coherence, without confirmed activity restriction [7,113]; 6. Social determinants: social isolation, inadequate caregiving or social support needs due to low income, loss of social role, or living alone, without evidence of home confinement [7,113]; 7. Environmental factors: stairs at home entrance, heavy doors, poor neighbourhood walkability, lack of transport access, or rural location, without confirmed home confinement statement linking them to functional impairment as jointly restricting access [7,120] (note: if the study reports that the combination of environmental factor and functional impairment restrict access, it is coded as PH6 instead). Insufficient/Not homebound-relevant: PH = 0 and ARH = 0 (decision made by reviewing the study). If the participant population does not present a risk consistent with homebound status and it is ambulatory without reported limitations, then “not homebound” is classified. If not, enough information provided in the study to code items, then the study is coded as “insufficient”. Notes: 1. When a study includes heterogeneous groups of participants (e.g., some are homebound or some are at risk or probable homebound), then both codes are assigned and the study is flagged as mixed in the extraction form. 2. An indicator was coded as a characteristic of the study population only when it applied to all or the great majority of participants, as reported in the eligibility criteria, the target-population description, or the baseline characteristics. Characteristics reported for smaller subgroups of participants were not attributed to the whole study population. Thresholds used in the coding framework and conventions The ≥2-indicator threshold for the code “Possibly Homebound” reflects a pattern observed among the subset of included studies where authors defined or described homebound status (Table 6 in the manuscript, *n* = 10; one descriptive study [85] was excluded from this because homebound status was implied rather than described, being inferred from the control group receiving the level of care typically provided to homebound people). Of the four studies applying a formal homebound definition, all combine at least two corroborating elements. For example, ADL dependency together with a leaving-home frequency limit, or difficulty leaving home together with the infrequency/short duration of absences, rather than relying on a single criterion. Of the six studies describing homebound status narratively, four of them likewise pair two elements, most often a functional/mobility descriptor (frailty, ADL/IADL dependency, motor or ambulation impairment) with a service-use or care-need descriptor (receipt of nursing or palliative care at home). This observation informed the decision to use two corroborating indicators before classifying an undeclared population as “Possibly Homebound” since a single mention of receiving “home-based service”, does not, on its own, confirm restriction to the home and neither does a single mobility-related ADL/IADL impairment. Requiring a second, independent indicator guards against this kind of false positive and increases the specificity of the “Possibly Homebound” classification. The single-indicator threshold retained for “At Risk of Homebound” preserves sensitivity for a category that is at risk rather than a confirmatory one. This pattern was derived only from the studies in Table 6 that already explicitly reported homebound status, and was then applied, as a pre-specified rule, to the separate subset of remaining studies where homebound status was not reported, avoiding circularity between the studies used to derive the threshold and those classified by it. In a sensitivity analysis, the PH threshold was moved to ≥1 and ≥3 indicators. Neither change moved any study between the homebound-relevant categories (Possibly Homebound, At Risk of Homebound) and not-homebound, as this distinction depends on the presence of any indicator rather than on their number. A ≥1 threshold would have classified 13 instead of 6 studies as Possibly Homebound, while a ≥3 threshold would have classified none, since no study reported more than two PH indicators. The ≥2 threshold was therefore retained as the most conservative rule, ensuring that the strongest classification “Possibly Homebound” was never assigned on weaker evidence. In a further sensitivity analysis, receipt of home-based services (PH4) was removed from the framework to test how much the Possibly Homebound category depended on this indicator. Five of the six Possibly Homebound studies [37,72,81,84,92] had PH4 as one of their two PH indicators and would therefore be classified as At Risk of Homebound. The sixth [94] met two other PH indicators and would remain Possibly Homebound. None of the studies would be classified as Not homebound, as all retained at least one other PH or ARH indicator.	
